# Supplementary material for: Chromothripsis during telomere crisis is independent of NHEJ, and consistent with a replicative origin
Source: Genome Res. 2019 May;29(5):737–49. doi: 10.1101/gr.240705.118 (PMC6499312; doi:10.1101/gr.240705.118)
Supplement: Supplemental Material [file supp_gr.240705.118_Supplemental_file_1.zip › contigs/annotated_contigs/DB114/contig.2.DB114_length_606_mean_cov_10.8910891089.docx]

**DB114_length_606_mean_cov_10.8910891089**

```
CATCAGTAGCAAGAGGAGGCAGGTGACACTACACATCTTCACCTGGAAACCCTTTTCTCCAACTGGCTGAAGTGGACCCGGGCTCCTGG
 >chr17:8133569-8133912 + E=1e-191
AAGTAGTCCTAGTGAGGGAGGCAAGTGTGGGTCTTCTATATATACATCCAGGTGAGGGGGGAATTCACATTCAGCAGTCTCAAGAGCGA

CTGTTAGCTTCACACACCTTCTCATGGCCCCCGTGTTCCCTATAGAAGGAAGGTGGGTGTTAGTAGGATCTGCGAAGTCCACAAAGTCC

CATCACTCTATTTCATACCTTCCCCACAAAGGAAGGGTCATTACCCAGTTTCATCCAGAGAGACGCCACAAGGGG|T|CTCACACATGG
                                                                            >chr17:8077
AGGAAAGGGCTGGGTAGGGGAAAAACCTTCAGGTTTAGGGCCAACCTTCCTCCCTCATCCCCAAATATTGGATGCTACCACACCTGCAG
742-8078006 + E=4e-147
AAGACAAAAATGTACCAATACGTAGTGCACTCCCAACGCTCGAATATGAAGAAGGACAGGGCCACGGATGCACTACAGTGAGCCCCAAT

GGCTTGGGAAGGCACGAGATTAAGGAAAGTCTTTGGATCGACAGACAGTGATGGGTCAGAAAAGGCCAGTGGTG
```
